# Individual and organizational resilience—Insights from healthcare providers in Germany during the COVID-19 pandemic

**DOI:** 10.3389/fpsyg.2022.965380

**Published:** 2022-08-25

**Authors:** Daniela Gröschke, Elisa Hofmann, Nadine D. Müller, Judith Wolf

**Affiliations:** Intercultural Human Resource Development and Organizational Development, Department of Intercultural Business Communication, Institute for German as a Foreign and Second Language and Intercultural Studies, Friedrich Schiller University, Jena, Germany

**Keywords:** COVID-19, healthcare providers, individual resilience, organizational resilience, team efficacy, transformation, team resilience, healthcare workers

## Abstract

We explored the effects of resilience in the healthcare setting during the COVID-19 pandemic in Germany. Our study sheds light on the cross-level effects of resilience in hospitals and thus responds to calls to research this empirically. In a cross-sectional study design, the perceptions of resilience of employees in hospitals and of transformations at the individual, team, and organizational level were analyzed. An online survey was conducted in summer 2020 in Germany in which 1,710 healthcare workers completed a self-report questionnaire. Results indicate that resilience is both a highly interrelated construct on the individual and organizational level and also positively linked to perceptions of transformation as an indicator for demonstration of resilience. We also found a partial mediation effect of organizational resilience and team efficacy, respectively, on the relationship between individual resilience and perceived transformation on the individual and organizational level as well as a full mediation on the team level. The study highlights the interdependence of individual and organizational resilience (which is mediated by team efficacy) and its impact on perceived transformation in German hospitals during the COVID-19 pandemic. Whereas team efficacy is crucial for performance in regular work operations, during a pandemic the organizational level becomes more relevant. Theoretical and practical implications are discussed.

## Introduction

Healthcare providers have played a critical role during the COVID-19 pandemic. Hospitals had to adjust their procedures and processes to respond to the pandemic (Sklar et al., 2021) and to function as a place of public safety. Popular press and academic literature reported about healthcare workers experiencing extraordinary challenges, such as feelings of stress and uncertainty (Lai et al., 2020; Shaukat et al., 2020; Vagni et al., 2020), the risk of testing positive for COVID-19 (Bandyopadhyay et al., 2020; Nguyen et al., 2020; Cohen and Nica, 2021), fear of infection, stigma, guilt, and social isolation (Banerjee et al., 2021), and depressive symptoms, emotional exhaustion, and psychological trauma symptoms (Mitchell and Lăzăroiu, 2021). Thus, the question arises, how employees in hospitals perceived and dealt with endeavors posed by the virus in their day-to-day work.

Resilience research offers a framework with which to understand the unique complexities in healthcare (Jeffcott et al., 2009). In various disciplines (for overviews, Bhamra et al., 2011; Hillmann and Guenther, 2021), resilience generally has been used to describe organizations, groups, or individuals that are able to react to and recover from stress or disturbances with minimal effects on stability and functioning (Sutcliffe and Vogus, 2003; Linnenluecke, 2017) as well as an adaptive capacity to bounce back, recover, and cope effectively with disturbance, stress, and adversity (for a recent overview on resilience definitions, see Raetze et al., 2021). More recently, the multilevel and multistage nature (Williams et al., 2017) of resilience has been highlighted but the concept still lacks clarity, especially regarding the interdependencies between individual, team, and organizational levels (Jeffcott et al., 2009). For example, a team of resilient members may not necessarily demonstrate high resilience at the group level because group interactions may lack clear communication or support (Alliger et al., 2015). Similarly, resilient individuals or teams might not directly build resilient organizations. Collective phenomena such as team or organizational resilience are hence not assumed to be just an additive composite of individual resilience (Lengnick-Hall et al., 2011)—but further processes are at play; on the contrary, highly resilient individuals might even be a barrier to a shared understanding in organizations (Horne and Orr, 1998). Therefore, resilience research needs to integrate findings across levels (Britt et al., 2016; Matheson et al., 2016; Robertson et al., 2016; Duchek, 2020) and to include the interaction between an organization, its stakeholders, and the environment during confrontations with adversity (Williams et al., 2017).

The purpose of this study was to build on and extend past research by empirically testing the interrelations of resilience during an adverse event: the COVID-19 pandemic. Specifically, we aimed to explore the effects of fostering resilience in the healthcare setting during the COVID-19 pandemic in Germany and sought to make four contributions to the literature:

First, empirical studies have addressed either the collective (organizational or team) or the individual level, leaving out the interplay between them. Conceptually, organizational resilience can be achieved through employees and teams. Hereby, individual, team, and organizational resilience are linked and influence each other reciprocally (Riolli and Savicki, 2003). For example, one suggestion has been that organizations can be only as resilient as their individuals are (Horne, 1997; Horne and Orr, 1998; Mallak, 1998; Coutu, 2002; Shin et al., 2012), but more holistic approaches (Lengnick-Hall et al., 2011) have proposed that individual resilience cannot simply be added up to reach organizational resilience. Similarly, one could expect that resilient organizations create an environment enabling individuals to show resilient behavior (Pangallo et al., 2015; Soucek et al., 2016; Wachs et al., 2016). Our empirical study helps clarify how the levels are related to each other. Recent research on the impact of COVID-19 on healthcare workers has addressed the negative effects on these workers (Benfante et al., 2020; Mhango et al., 2020; Couarraze et al., 2021; Riguzzi and Gashi, 2021), the personal resources at the individual level (e.g., Fino et al., 2021), or both (Coulombe et al., 2020; Huffman et al., 2021; Jo et al., 2021) but has barely touched on supportive factors at the collective levels (Labrague and de los Santos, 2020; Tam et al., 2021). We contribute to this literature by providing insights on how organizational- and team-level facets interact with individual facets.

Second, this study addresses resilience in terms of responses and reactions during a specific adverse event, namely, the COVID-19 pandemic. Resilience has been studied in relation to several events (for a review, see Linnenluecke, 2017) that have also assumed that resilience differs according to the nature of the adversity (Martin-Breen and Anderies, 2011). We examined the processes of resilience during the COVID-19 pandemic in hospitals instead of examining resilience before or after an adverse event.

Third, we have followed the advice of Britt et al. (2016) to differentiate between resources for resilience and demonstrations of resilience. We investigated the connections between resources for resilience and perceptions of transformation as demonstration of resilience. Hence, we contribute to the literature by proposing perceptions of transformation as a promising measure for positive adaptation, i.e., an outcome of resilience. We assumed that resilience levels would affect how employees perceived transformations due to the COVID-19 pandemic and we expected that higher levels of individual and collective resilience would lead to more positive perceptions of transformation, which could be interpreted as adaptations that are more positive. By perceptions of transformation we mean specific aspects of life that have been affected by the COVID-19 pandemic at the individual (e.g., work–life balance) and collective (e.g., skills and competencies in the team, communication in the organization across departments) levels. Thus, we have expanded existing research by highlighting individual-, team-, and organizational-level resources for maintaining positive functioning during COVID-19.

Fourth, our study was conducted in the healthcare sector in Germany. Resilience research in this sector has been explored mainly in East-Asian, African, and Arab countries, as these are places associated with higher population density and higher risk of disasters, and many have already faced other epidemic events such as SARS, Ebola, and MERS (Koh et al., 2005; Wong et al., 2008; Khalid et al., 2016; Jalloh et al., 2017). Germany has been a country with less experience in epidemic and pandemic outbreaks or natural disasters. Although recent studies and reviews on the COVID-19 pandemic have investigated resilience in healthcare workers in different countries (see, e.g., Bozdağ and Ergün, 2021; Di Trani et al., 2021; Douillet et al., 2021; Rieckert et al., 2021), studies in Germany are still lacking. Further, we focused our analysis on hospitals as key players in the healthcare system and integrated various occupation types within a hospital in our survey. Key participants in research in the healthcare sector are medical staff (doctors, nurses), whereas administrative and other service staff members (facility management, cleaning) are often neglected. Understanding hospitals as a system, one can gain deeper knowledge of resilience processes by integrating relevant key stakeholders. This approach broadens the understanding of resilience in a healthcare organization.

## Background and hypothesized model

A crisis such as the COVID-19 pandemic disrupts normal operations and creates emergent job demands in a context characterized by urgency, uncertainty, and threat (Sayegh et al., 2004). Therefore, resilience resources should be activated to maintain normal functioning at individual and collective levels within hospitals and over a longer period. The capacity for resilience (resilience resources) addresses the personal and collective factors associated with the ability to show or likelihood of showing positive adaptation in the face of significant adversity, whereas the demonstration of resilience refers to the documentation of positive adaptation (Britt et al., 2016). Thus, resilience at the different levels is expected to influence healthcare workers’ perceptions of transformation on the individual, team, and organizational level.

### Resilience at the individual level

There is no universally accepted definition of resilience in the empirical literature published this century, however key markers of resilience are: rising above to overcome adversity, adaptation and adjustment, ‘ordinary magic’, good mental health as a proxy for resilience, and the ability to bounce back (Aburn et al., 2016). Other common characteristics of resilient individuals are their recognition of the need for a firm acceptance of reality, virtue, and the deep belief that life is meaningful, as well as the ability to improvise and adapt to significant change (Coutu, 2002).

Individual resilience is conceptualized as a trait, capacity, or process. The trait perspective understands psychological resilience as the ability to emotionally cope with a crisis, allowing the person to return to the precritical state and thus to promote personal assets and protect the self from the potential negative effects of stressors (Masten, 2001; de Terte and Stephens, 2014). The capacity concept sees resilience as ‘psychological capital’ that helps a person manage stressors and losses and to engage higher state-like psychological resource capacities by means of humor, hope, self-efficacy, and optimism (e.g., Luthans and Youssef, 2007). The process approach sees resilience as a ‘fluid process’ rather than a dichotomous construct (Werner and Smith, 1979). In this perspective, resilience is a dynamic process encompassing positive adaptation in the context of significant adversity (Hartmann et al., 2020).

Regarding COVID-19, Banerjee et al. (2021) used a qualitative approach to gain deeper insights into the dynamic processes of resilience and to describe how healthcare workers used their resilience to navigate through adverse situations in Indian hospitals. Healthcare workers formed a ‘resilient identity’ by harnessing social support, rooted in morality, gratitude, and a sense of purpose. They managed the resilience by applying stress-management strategies (e.g., regular dialogue with themselves, decreasing expectations, promoting self-care, and reducing self-stigma) and working through the socio-occupational distress by self-commitment and care (adequate sleep, diet, hobbies, small celebrations, festivities, etc.). Another review highlighted that coping behaviors, resilience, and social support were associated with positive mental and psychological health outcomes (Labrague, 2021).

In this paper we have conceptualized individual resilience as a capacity that enables healthcare workers to maintain functioning during the COVID-19 pandemic, resulting in positive adaptation and learning. Hence, we have also perceived resilience itself as a dynamic process that includes resilience capacities and respective resilience outcomes in the case of an activation.

### Resilience at the organizational level

Resilience at the organizational level is conceptually different from that at the individual level (Lengnick-Hall et al., 2011; Carmeli et al., 2013; Bowers et al., 2017; Morgan et al., 2017). Organizational resilience has also been defined in many ways, for instance, as a capability, capacity, characteristic, outcome, process, behavior, strategy or approach, and type of performance, or as a mix of these (Hillmann and Guenther, 2021). In a comprehensive understanding, resilient organizations promote competence, restore efficacy, and encourage growth through the behavioral processes of mindful organizing enacted by frontline employees (Vogus and Sutcliffe, 2007). There is agreement that organizational resilience develops over time (Vogus and Sutcliffe, 2007; Hillmann and Guenther, 2021), and that every organization has its own way to achieve resilience; thus there is no magic 10-step formula (Horne, 1997). Hillmann and Guenther (2021) concluded that organizational resilience is the ability of an organization to maintain functions and recover fast from adversity by mobilizing and accessing the resources needed. An organization’s resilient behavior, resilience resources, and resilience capabilities thus enable and determine organizational resilience. The idea that resilience is commonplace and required across organization types shows up in the organizational literature as well (Williams et al., 2017).

Empirical research on organizational resilience is still sparse in terms of providing a valid measurement scale for organizational resilience (Mallak, 1998; Pal et al., 2014; Richtnér and Löfsten, 2014; e.g., Barasa et al., 2018). For the healthcare sector, Mallak (1998) identified six variables describing resilience: goal-directed solution seeking; avoidance; critical understanding; role dependence; source reliance; and resource access. Jeffcott et al. (2009), following Wreathall (2006), conceptualized organizational resilience in the healthcare sector as a set of seven factors: top-level commitment, just culture, learning culture, awareness, preparedness, flexibility, and opacity. Resilience as a process further includes multiple stages over time. Anticipating, coping, and adaptation (Duchek, 2020) should be seen as demonstration of resilient behavior.

Similar to individual resilience, organizational resilience as we understand it is both a capacity and a process. If capacities of organizational resilience are activated, they support healthcare providers and their employees in maintaining functioning during the COVID-19 pandemic. Taking a process perspective, this in turn leads to positive perceptions of transformation as an outcome of resilience.

### Interconnection of individual and organizational resilience

The literature on the interconnection of individual and organizational resilience is more conceptual and still sparse (see, for reviews, Hartmann et al., 2020; Raetze et al., 2021). In general, organizational resilience can be seen as an important context characteristic that fosters individual resilience. Previous empirical research has focused on one or more facets of organizational resilience in relation to other variables and not on organizational resilience as a holistic construct. Research on programs fostering resilience in organizations have highlighted that organizational resilience affects individual resilience (e.g., Teng-Calleja et al., 2020). Considering time issues, Prayag et al. (2020) found that individual resilience (demonstrated as life satisfaction) increased organizational resilience of entrepreneurs. In the context of COVID-19, in a study among 69 frontline healthcare providers in China, Tam et al. (2021) highlighted the lack of institutional supportive responses to COVID-19 as a direct source of distress for the employees. Moreover, they found support of positive effects of institutional support on individual resilience and lower psychological distress of healthcare workers in face of COVID-19 stressors. Thus, institutions play a critical role in providing support for healthcare providers. In a similar vein, Labrague and de los Santos (2020) investigated the interaction between organizational support, social support, and individual resilience for nurses in the Philippines during the COVID-19 pandemic. Their results indicate that nurses can show higher levels of resilience when organizational and social support exist. In line with these findings, Matheson et al. (2016) put forward the idea that the work environment in the healthcare sector needs to be in alignment with individual resilience. There is some evidence that an institutional variable such as an organizational safety culture leads to better team performance (Heckemann et al., 2019). Gonçalves et al. (2022) emphasized that organizational resilience is an important factor in how healthcare workers perceive stress and adapt to work-related challenges. Given the literature on individual and organizational resilience, we developed our first hypothesis:

*Hypothesis 1*: Individual and organizational resilience are positively related to each other.

### Resilience and efficacy at the team level

The team-stress literature highlights that adverse events cause stress in teams, which has deleterious effects (Driskell and Salas, 1991). For example, in situations of high occupational stressors, most individuals perceive psychological strain, focusing inward and losing focus on the team task as well as on the interdependencies within the team. External threats significantly reduce the communication channels available to and amount of information used by team members (Gladstein and Reilly, 1985). This, in turn, inhibits team satisfaction and increases the potential for conflict because of miscommunication and poor role coordination. Similarly, research on team resilience has assumed that resilient teams can resist the negative impact of adverse events by showing minimal disruption to their performance (Hartwig et al., 2020).

Team resilience as a positive team-level capacity refers to processes of “managing pressure effectively across the team as a whole […] that further strengthen the capacity of the team to deal with future challenges in adversity” (Flint-Taylor and Cooper, 2017). A recent review referred to team resilience as “an emergent state resulting from resilient team processes, which are fostered by team composition and contextual factors” (Gucciardi et al., 2018; Hartwig et al., 2020) and even as a “second-order emergent state that is actually the result of other emergent states in the team” (Bowers et al., 2017). The assumption here is that team resilience mediates other team emergent states and outcomes during times of stress. One of those first-order emergent states is team efficacy. Team efficacy and team resilience are somehow related (Bowers et al., 2017), and some researchers have used the terms team resilience and team efficacy interchangeably (McCray et al., 2016). Both need time to build up through team interactions, and then they relate to important team outcomes (e.g., Chen et al., 2005). Conceptual unclarity also exists when measuring team resilience, for example, by integrating (Sharma and Sharma, 2016) or not integrating (McEwen and Boyd, 2018) team efficacy. However, as theoretical conceptualizations of team resilience often revolve around team efficacy, in this paper we apply team efficacy as a proxy for team resilience.

Team efficacy (also known as collective efficacy) refers to the belief that the team has the ability to perform the job tasks successfully (Lindsley et al., 1995; Bandura, 2000). Team efficacy (as a first-order emergent state) has received far more attention because of increased team-based structures in organizations. Thus, more conceptual clarity and empirical evidence exists regarding team efficacy. Especially in the healthcare context, self-efficacy and team efficacy have been researched in depth. High team efficacy has been associated with decreased burnout of nurses (Zellars et al., 1999) and higher satisfaction and commitment, as well as buffering the stressor–strain relations (Jex and Bliese, 1999). Furthermore, high team efficacy has been related to increased cooperation and an atmosphere of meaningful interpersonal relationships (Lee and Ko, 2010) and reduced missed care (Duffy et al., 2018; Smith et al., 2018). In contexts of high interdependence, team efficacy has been closely related to performance (Gully et al., 2002) and to change-related issues such as the perception of cohesion (e.g., Morgan et al., 2019). Team efficacy also functions as a mediator between transformational leadership and well-being (Nielsen et al., 2009) as well as between work stressors and burnout (Day et al., 2009). Thus, nurse performance was found to be highly dependent on contextual variables such as collective efficacy, leadership style, or unit culture (Lee and Ko, 2010) but also on resources such as workload, staffing, and implicit rationing (Zhao et al., 2020).

Team efficacy can be seen as a protective factor that increases individual resilience in the workplace. Resilient team members have a comprehensive understanding of team processes, team goals, and objectives, and they discuss team-member roles to guide each other’s actions (Mallak and Yildiz, 2016). Especially in a crisis, team efficacy has bearing in the ability and motivation of both the team as a whole and each individual team member. Gichuhi (2021), for instance, emphasized that during a crisis, collaboration is critical to empower and support teams’ efforts to confront the day’s challenges in a constructive way and to maximize team efficacy. In this line, Traylor et al. (2021) refer to the importance of collective efficacy for frontline healthcare workers because a lack of experience with COVID-19 might reduce team members’ believe to be successful in treating patients. First empirical insights during COVID-19 highlight that collective efficacy is a significant predictor of risk perception, which relates to adaptation of preventive health behavior across 10 countries (Dryhurst et al., 2020). In the Italian healthcare sector, physicians’ collective efficacy beliefs and sense of belonging to their hospital were positively associated with job satisfaction (Capone et al., 2022).

In summary, we conceptualized team efficacy as a capacity that can be activated during a crisis and in turn leads to a positive resilience outcome. Further, we concluded that team efficacy has positively impacted the resilience of healthcare providers and their workers during COVID-19. Hence,

*Hypothesis 2*: Team efficacy is positively related to both individual and organizational resilience.

### Perceptions of transformation as a demonstration of resilience

Research is still quite inconsistent on defining what is meant by ‘positive adaptation’ when demonstrating resilience. One main approach is to conceptualize positive adaptation resulting in growth and learning as a potential outcome of resilience. Britt et al. (2016) proposed four categories that demonstrate individual resilience: job performance, low stress symptoms, high well-being, and healthy relationships. Other researchers have endorsed resilience as an adaptive capacity to modify or change to cope better with stressors (Kärner et al., 2021). The underlying assumption is that an employee’s attitude toward the process of transformation is determined by the resilience and adaptive capacity of a system. From the perspective of job demands–resources models, resilience as a personal resource acts as a buffer against the negative influence of work demands (Martinez-Corts et al., 2015; e.g., de Clercq and Belausteguigoitia, 2017). From the perspective of conservation of resources theory, resilience can help individuals obtain additional resources from the environment (e.g., Shin et al., 2012). In the COVID-19 context, resources for reducing stress and increasing job satisfaction are for example internal organizational communication, employee reward systems, and skills capitalization (Nemțeanu et al., 2022). Overall, individual resilience has been found to be indirectly related to job performance, organizational citizenship behavior, and career success and directly related to job satisfaction (Larson and Luthans, 2006; Youssef and Luthans, 2007), mental and physical health (e.g., burnout, emotional exhaustion, biopsychological distress; Soucek et al., 2016), healthy relationships, and change-related and work-related attitudes (e.g., psychological contract awareness, happiness; Hartmann et al., 2020). On an organizational level, the result of an organization’s response to adversity is positive adaption as well as growth and learning.

In this paper, we consider perceptions of transformation on the individual, team, and organizational level as a demonstration of resilience. In line with Martinez-Corts et al. (2015), we understand resilience as being “related to a more positive appraisal of stressful situations and the use of more active and approach-related coping” (p. 328) and expect that activated resilience is expressed in the fact that one tends to perceive and evaluate transformation more positively because of the opportunities for learning and growth. Thus we emphasize such a connection between resilience and perceptions of transformation for the individual and collective level. Hence,

*Hypothesis 3*: Resilience, as experienced by hospital employees, leads to positive perceptions of transformation at different levels in healthcare providers during the COVID-19 pandemic.

*Hypothesis 3a*: Individual resilience is positively related to perceptions of transformation at the individual, team, and organizational level.

*Hypothesis 3b*: Team efficacy is positively related to perceptions of transformation at the individual, team, and organizational level.

*Hypothesis 3c*: Organizational resilience is positively related to perceptions of transformation at the individual, team, and organizational level.

**Figure 1:**
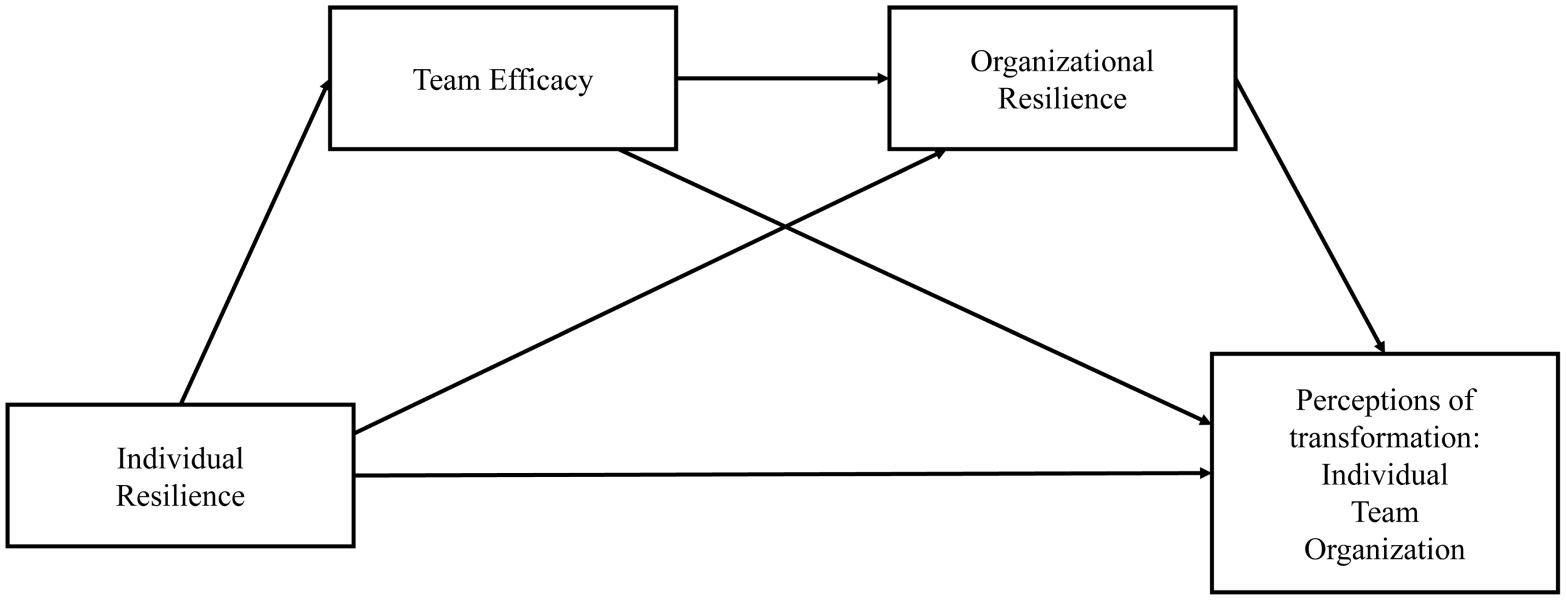
Proposed research model: sequential mediation.

Our research model is outlined in Figure 1: Resilient behavior of individuals, teams, and organizations is required to effectively manage and overcome a pandemic event such as COVID-19. These levels are interlinked and of a dynamic nature. As resilient teams and organizations are more than the sum of resilient individuals, resilience of organizations should mediate the positive link between individual resilience and positive outcomes at all levels. Team efficacy as a protective factor for workplace resilience (Sharma and Sharma, 2016) should mediate the relationship between individual and organizational resilience. Avanzi et al. (2015) found a mediating effect of team efficacy relating to lower burnout (for teachers). In other words, organizational resilience provides the context for fostering team efficacy. High team efficacy enables resilient behavior of individuals in hospitals. Thus, we assume that employees are only able to show positive adaptation when organizational resilience processes are in place and high team efficacy is present. In a context supporting team efficacy and individual resilience, transformation will be evaluated more positively. Accordingly, we predict

*Hypothesis 4*: Organizational resilience mediates the relationship between individual resilience and positive perceptions of transformation at the individual, team, and organizational level.

*Hypothesis 5*: Team efficacy mediates the relationship between individual resilience and positive perceptions of transformation at the individual, team, and organizational level.

## Materials and methods

### Design

An online survey based on a cross-sectional design was conducted from July 6, 2020, to October 13, 2020, in Germany. To obtain timely insights on the experience and behavior of hospital employees, we recruited a so-called convenience sample, which was assembled according to the snowball principle. Initial contacts were acquired through the research project network as well as through internet research on associations, institutions, and organizations in the hospital context. In addition, the link to the survey was forwarded directly to hospitals *via* a large central German organization. Participants needed 25 min on average to complete the survey.^1^ The survey was distributed *via* SoSci Survey and was formulated in German. Response anonymity was ensured.

### Participants

In total, 1,730 individuals completed the online questionnaire; after cleaning, 1,710 were included in the analysis (20 participants were excluded from the data analysis owing to inconclusive responses, too many missing values, and response durations being too short). A detailed overview of the sample and descriptive statistics is presented in Table A1 in the Appendix.

The majority (70%, *n* = 1,192) were women and nearly 30% (*n* = 504) were men. Our sample covered various ages: 21% (*n* = 360) of participants were between 18 and 32 years old, 36% (*n* = 621) were between 33 and 47 years old, 40% (*n* = 675) were between 48 and 62 years old, and 2% (*n* = 34) were over 62 years old. About a third (34%) of respondents had responsibility for others in their own household. Participants in our sample spanned a broad variety of occupations: 37% worked as nurses, 14% as doctors, 16% as medical support, 28% in administration, and 5% in other areas. The majority of participants (54%, *n* = 918) had already completed pandemic training.

### Measures

We mainly used short versions of scales to fit the busy schedules of healthcare workers during the ongoing pandemic. Most of the scales were developed and pretested in German in a preliminary unpublished study on preparation for an endemic scenario (manuscript currently in preparation). The study focused on individual perceptions (of individual and organizational resilience and team efficacy) and individual outcomes (perceptions of transformation as demonstration of resilience). The measures are described in detail in the following and will be provided by the authors on request.

#### Individual resilience

We elicited individual resilience by measuring it with the Resilient Behavior at Work short scale adapted from Soucek et al. (2015). The short scale contains eight items (e.g., “When faced with difficult tasks at work, I kept my eyes on my goal and did not allow myself to be diverted from my path”), rated on a 6-point Likert scale ranging from 0 (does not apply at all) to 5 (fully applies). Our data indicated good internal consistency (*α* = 0.80).

#### Team efficacy

Team efficacy was measured with the respective subscale of the Team Resilience Scale (Sharma and Sharma, 2016). The team efficacy subscale has nine items (e.g., “I trusted that my team could handle such a situation well”), rated on a 6-point Likert scale ranging from 0 (does not apply at all) to 5 (fully applies). The internal consistency of the scale was very high (*α* = 0.96).

#### Organizational resilience

In a preliminary study, a scale of organizational resilience was developed, based on Mallak (1998), Jeffcott et al. (2009), Toner et al. (2017), and Organizational Resilience Health Check (2019) and tested in two partner hospitals. Out of this scale and based on measurement metrics of the pre-study, seven items were chosen covering opacity, flexibility, learning culture, preparedness, top-level commitment, awareness, and just culture. Participants rated the items (e.g., “Contingency planning included the potential impact on employees and the team”) on a 5-point scale ranging from 0 (do not agree at all) to 4 (fully agree). Internal consistency of the Organizational Resilience short scale was high (*α* = 0.84).

#### Perceptions of transformation

We elicited perceptions of transformation during the COVID-19 pandemic on the individual, team, and organizational level as a dependent variable and as an indicator for the demonstration of resilience (Britt et al., 2016). Participants were asked to indicate on 5-point Likert-type scales to what degree specific aspects on each level, respectively, had been worsened (1), were unchanged (3), or had been improved (5) compared to before the COVID-19 pandemic. Internal consistencies of the three scales were low on the individual level (*α* = 0.67) and good on the team level (*α* = 0.79) and the organizational level (*α* = 0.80).

#### Control variables

As control variables (see Table A1 in the Appendix), we measured sociodemographic variables as well as some COVID-19-specific variables. As sociodemographic variables, we measured age, sex, occupation type, care responsibilities in participants’ own household (e.g., elder care or childcare), and worries about the lack of (child-)care. For COVID-19-specific control variables, we elicited perceived risk of infection and whether participants had completed pandemic training on the transmission routes of highly contagious diseases and how to use personal protection equipment properly.

## Results

### Reducing common method bias

To reduce common method bias (Podsakoff et al., 2003), we addressed item context effects by randomly assigning the items. Thus, counterbalancing the item order helps control for priming effects. Item characteristic effects were reduced by incorporating different scale formats and scale anchors. Different response formats were chosen for predictor and criterion variables also to control for acquiescence bias.

As a second approach to reduce common method bias, we conducted a Harman’s single factor test using principal axis factoring including all 24 items of the constructs individual resilience, team efficacy, and organizational resilience. The Kaiser–Meyer–Olkin measure of sampling adequacy was 0.95 and the Bartlett’s test of sphericity was significant (*p* < 0.001). No single factor accounted for more than 50% of the variance, hence the factor loadings are all below the recommended 50% threshold (Podsakoff et al., 2003). An exploratory factor analysis revealed a four-factor solution (team efficacy, organizational resilience, individual resilience 1, individual resilience 2), where the latter two factors were subfactors of one construct (individual resilience). The first factor (team efficacy, nine items) accounted for 37% of the variance, the second factor (organizational resilience, seven items) for 9% of the variance, the third factor (individual resilience 1, five items) for 7% of the variance, and the fourth factor (individual resilience 2, three items) for 2% of the variance.

We further conducted a confirmatory factor analysis using principal axis factoring relating each of the items to their respective theoretical constructs (team efficacy, organizational resilience, individual resilience). Factor 1 (team efficacy) comprised nine items and explained 37% of the overall variance. Factor loadings ranged from 0.597 to 0.880. Factor 2 (organizational resilience) contained seven items and explained 9% of the overall variance with factor loadings from 0.372 to 0.738. Factor 3 (individual resilience) contained eight items and accounted for 7% of the overall variance with factor loadings from 0.478 to 0.671. The theoretically driven three-factor solution accounted for 53% of the overall variance. We compared the three-factor model to a next-most-likely four-factor model (55% explained variance) and a single-factor model (accounting for 37% of the explained variance). The three-factor solution resulted in the second-highest explanation of variances and was in line with our theoretical assumptions.

### Hypotheses testing

All results were calculated with IBM SPSS Statistics 27. To test our mediation hypotheses and research models, we used the SPSS PROCESS macro by Hayes (2017). First, we analyzed the hypotheses regarding the relationships between individual resilience, team efficacy, and organizational resilience (Hypotheses 1 and 2). Second, we focused on their relationships with their respective counterparts of the transformation variables (Hypotheses 3 and 3a–c). Finally, we tested the hypotheses regarding the proposed sequential mediation models (Hypotheses 4 and 5) by applying model 6 from the SPSS PROCESS macro (X = predictor; Y = outcome; M1 and M2 = mediators). We included individual resilience as X, perceived transformations on the individual, team, and organizational level as Ys, team efficacy as M1, and organizational resilience as M2. Table 1 summarizes the correlations among the independent and dependent variables.

**Table 1:** Descriptive statistics and correlations of the measures.

Variable	Cronbach’s *α*	M (SD)	1	2	3	4	5	6
1. Individual resilience	0.800 (8 items)	3.63 (0.83)	–					
2. Team efficacy	0.957 (9 items)	3.83 (1.00)	0.39^***^	–				
3. Organizational resilience	0.837 (7 items)	2.63 (0.82)	0.36^***^	0.52^***^	–			
4. Individual transformation	0.669 (3 items)	2.75 (0.63)	0.30^***^	0.27^***^	0.34^***^	–		
5. Team transformation	0.785 (4 items)	3.23 (0.56)	0.24^***^	0.41^***^	0.38^***^	0.33^***^	–	
6. Organizational transformation	0.796 (5 items)	3.07 (0.64)	0.27^***^	0.32^***^	0.59^***^	0.46^***^	0.54^***^	–

Scales ranged from 0 to 5 for individual resilience and team efficacy, from 0 to 4 for organizational resilience, and from 1 to 5 for individual transformation, team transformation, and organizational transformation. M, mean; SD, standard deviation.

Significant at level ***p < 0.001.

Correlations among variables were in line with our expectations. Individual resilience is positively related to organizational resilience (*r* = 0.36, *p* < 0.001) supporting Hypothesis 1. Further, team efficacy is positively related to individual resilience (*r* = 0.39, *p* < 0.001) and organizational resilience (*r* = 0.52, *p* < 0.001), supporting Hypothesis 2. Finally, in line with our Hypothesis 3, there are positive relationships between individual resilience, team efficacy, and organizational resilience, respectively, and perceptions of transformation. The correlation analysis indicated a positive relationship for the individual level (*r* = 0.30, *p* < 0.001), the team level (*r* = 0.41, *p* < 0.001), and the organizational level (*r* = 0.59, *p* < 0.001). Interestingly, participants perceived variables concerning the organizational level more strongly associated than variables on the team level. During the COVID-19 pandemic, organizational resilience of hospitals thus seems to have been a crucial factor in successfully responding to the pandemic as an adverse event. The results from the correlation analysis were further confirmed by multiple linear regressions. Table 2 provides the estimated regression results. Individual resilience, team efficacy, and organizational resilience were added as explanatory variables. The dependent variable varied in the three models. Individual transformation was the dependent variable in Model 1, and team transformation was the dependent variable in Model 2. Model 3 included organizational transformation as dependent variable.

**Table 2:** Individual resilience, team efficacy, and organizational resilience as determinants of perceptions of transformation.

Variable	Model 1 (DV = Individual transformation)	Model 2 (DV = Team transformation)	Model 3 (DV = Organizational transformation)
Individual resilience	0.18^∗∗∗^	0.05^∗^	0.06^∗^
Team efficacy	0.09^∗∗^	0.27^∗∗∗^	0.001
Organizational resilience	0.23^∗∗∗^	0.23^∗∗∗^	0.58^∗∗∗^
Observations	1,682	1,681	1,682
*R* ^2^	0.16	0.21	0.36
Adjusted *R*^2^	0.15	0.21	0.36

Regression results (standardized beta coefficients) from multiple linear regression models. Individual transformation was the dependent variable in Model 1, team transformation in Model 2, and organizational transformation in Model 3. DV, dependent variable.

Significant at level *p < 0.05, **p < 0.01, ***p < 0.001.

In all specifications, we found positive and significant main effects of individual resilience and organizational resilience on perceptions of individual, team, and organizational transformation. That is, perceptions of transformation were perceived as more positive the higher the levels of individual resilience, team efficacy, and organizational resilience were perceived by the study participants. In Model 1, we find that perceptions of transformations on the individual level were significantly predicted by individual resilience [*β* = 0.18, *t*(1679) = 7.34, *p* < 0.001], team efficacy [*β* = 0.09, *t*(1679) = 3.16, *p* = 0.002], and organizational resilience [*β* = 0.23, *t*(1679) = 8.51, *p* < 0.001]. In Model 2, we find that perceptions of transformations on the team level were significantly predicted by individual resilience [*β* = 0.05, *t*(1678) = 2.07, *p* = 0.039], team efficacy [*β* = 0.27, *t*(1678) = 10.47, *p* < 0.001], and organizational resilience [*β* = 0.23, *t*(1678) = 8.84, *p* < 0.001]. In Model 3, we find that perceptions of transformations on the organizational level were significantly predicted by individual resilience [*β* = 0.06, *t*(1679) = 2.54, *p* = 0.011] and organizational resilience [*β* = 0.58, *t*(1679) = 24.60, *p* < 0.001] but not by team efficacy [*β* = 0.001, *t*(1679) = 0.05, *p* = 0.959]. Organizational resilience had the largest impact on individual and organizational transformation perceptions and the second largest impact on team transformation processes. Team efficacy was a significant predictor of perceptions of transformation on the individual and team level but not on the organizational level. This result underscores the relevance of organizational resilience in perceptions of transformation during a crisis: Team efficacy and organizational resilience predicted perceptions of transformation beyond individual resilience. Analyses for multicollinearity reveal variance inflation factors (VIF) below 2, indicating no multicollinearity among the variables. Model 3 explained the largest amount of variance [*R*^2^ = 0.36, adjusted *R*^2^ = 0.36, *F*(3,1,679) = 311.76, *p* < 0.001), followed by Model 2 (*R*^2^ = 0.21, adjusted *R*^2^ = 0.21, *F*(3,1,678) = 151.31, *p* < 0.001), and last, Model 1 (*R*^2^ = 0.16, adjusted *R*^2^ = 0.15, *F*(3,1,679) = 102.85, *p* < 0.001). The regression results remained similar when control variables were included. The regression results with control variables are reported in Table A2 in the Appendix. This result provides further evidence for the importance of organizational resilience during a crisis. Hence, Hypotheses 3a, 3b, and 3c were supported.

**Figure 2:**
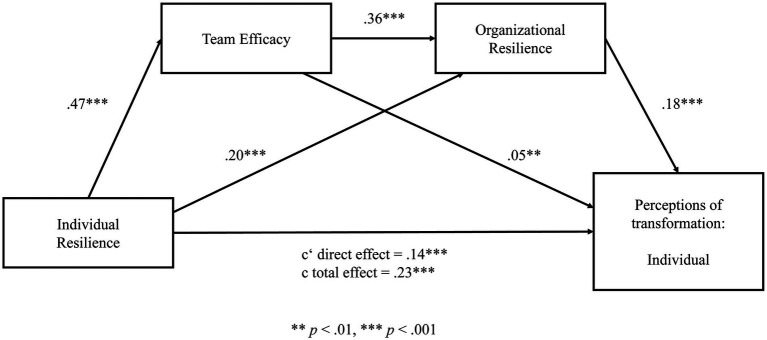
Sequential mediation model for perceptions of transformation on the individual level. Significant at level **p < 0.01, ***p < 0.001.

**Figure 3:**
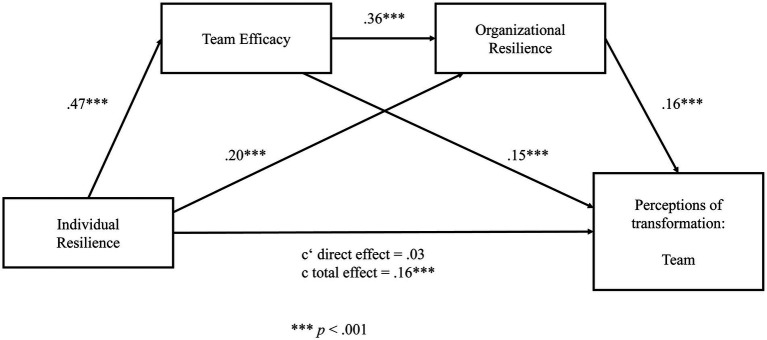
Sequential mediation model for perceptions of transformation on the team level. Significant at level ***p < 0.001.

**Figure 4:**
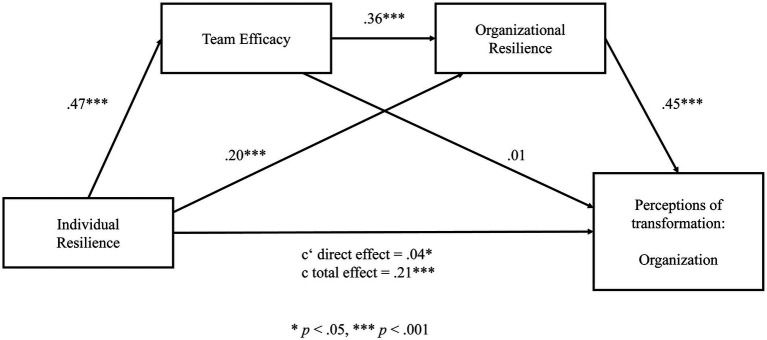
Sequential mediation model for perceptions of transformation on the organizational level. Significant at level *p < 0.05, ***p < 0.001.

Hypotheses 4 and 5 predicted a sequential mediation of team efficacy and organizational resilience between individual resilience and perceptions of transformation on the individual, team, and organizational level. Three sequential mediation models for each level were run, respectively, to test these hypotheses. The results of the path models are illustrated in Figure 2 for the individual level, in Figure 3 for the team level, and in Figure 4 for the organizational level. Detailed results of the mediation analyses are reported in Tables A3-A5 in the Appendix.

We first aimed to test whether team efficacy and organizational resilience mediates the relationship between individual resilience and perceptions of individual transformation. In a first step, the results reveal a significant total effect (c) of the predictor (individual resilience) on the outcome (perceptions of individual transformation); *β* = 0.23, *t* = 12.05, *p* < 0.001. Also, the total direct effect (c’) without the effect of the two mediators was significant (*β* = 0.14, *t* = 7.01, *p* < 0.001). In a second step, data analysis reveals that individual resilience significantly predicts team efficacy (*β* = 0.47, *t* = 14.59, *p* < 0.001) and organizational resilience (*β* = 0.20, *t* = 8.11, *p* < 0.001). Further, team efficacy significantly predicts organizational resilience (*β* = 0.36, *t* = 17.90, *p* < 0.001). In a third step, the results reveal that team efficacy (*β* = 0.05, *t* = 2.80, *p* = 0.005) and organizational resilience significantly predict perceptions on individual transformation (*β* = 0.18, *t* = 7.81, *p* < 0.001). In order to estimate the significance of the total indirect effect, we calculated 95% confidence intervals using 10.000 bootstrap resamples. The value “0″ was not contained in the interval, thus we can conclude that the indirect effect is significant; 95% CI (0.02, 0.04).

We ran an identical mediation analysis for perceptions of team transformation. In a first step, the results reveal a significant total effect (c) of the predictor (individual resilience) on the outcome (perceptions of team transformation; *β* = 0.16, *t* = 9.46, *p* < 0.001). The total direct effect (c’) without the effect of the two mediators was not significant (*β* = 0.03, *t* = 1.95, *p* = 0.051). In a second step, data analysis reveals that individual resilience significantly predicts team efficacy (*β* = 0.47, *t* = 14.59, *p* < 0.001) and organizational resilience (*β* = 0.20, *t* = 8.10, *p* < 0.001). Further, team efficacy significantly predicts organizational resilience (*β* = 0.36, *t* = 17.90, *p* < 0.001). In a third step, the results show that team efficacy (*β* = 0.15, *t* = 9.11, *p* < 0.001) and organizational resilience significantly predict perceptions on team transformation (*β* = 0.16, *t* = 7.64, *p* < 0.001). In order to estimate the significance of the total indirect effect, we calculated 95% confidence intervals using 10.000 bootstrap resamples. As the value “0″ was not contained in the interval, we can conclude that the indirect effect is significant; 95% CI (0.02, 0.04).

Lastly, we ran a mediation analysis for perceptions of organizational transformation. In a first step, the results reveal a significant total effect (c) of the predictor (individual resilience) on the outcome (perceptions of organizational transformation; *β* = 0.21, *t* = 10.84, *p* < 0.001). Also, the total direct effect (c’) without the effect of the two mediators was significant (*β* = 0.04, *t* = 2.52, *p* = 0.012). In a second step, data analysis reveals that individual resilience significantly predicts team efficacy (*β* = 0.47, *t* = 14.58, *p* < 0.001) and organizational resilience (*β* = 0.20, *t* = 8.10, *p* < 0.001). Further, team efficacy significantly predicts organizational resilience (*β* = 0.36, *t* = 17.93, *p* < 0.001). In a third step, the results show that organizational resilience (*β* = 0.45, *t* = 22.63, *p* < 0.001) but not team efficacy (*β* = 0.01, *t* = 0.05, *p* = 0.961) significantly predicts perceptions on organizational transformation. In order to estimate the significance of the total indirect effect, we calculated 95% confidence intervals using 10.000 bootstrap resamples. The value “0″ was not contained in the interval, thus we can conclude that the indirect effect is significant; 95% CI (0.06, 0.09).

On the individual and organizational level, a partial sequential mediation effect of organizational resilience was found. There was a significant indirect effect of individual resilience on perceptions of individual and organizational transformation through organizational resilience, a significant direct effect of individual resilience on perceptions of individual and organizational transformation, and a significant total effect. For the team level, we find a full mediation. While the indirect effect and the total effect are significant, the direct effect of individual resilience on organizational transformation remains insignificant. Hence our results indicate empirical evidence in support of Hypothesis 4.

Team efficacy partially mediated the relationship between individual resilience and individual transformation and fully mediated the relationship between individual resilience and team transformation but not organizational transformation. Hence, Hypothesis 5 can be only partly confirmed.

Overall, individual resilience relates to more positive perceptions of transformation at different levels in hospitals through consecutive mediating steps—*via* enhanced team efficacy and higher organizational resilience.

## Discussion

### Key findings

This study aimed to shed light on the cross-level effects of resilience in hospitals and has thus responded to calls to research this topic empirically (Jeffcott et al., 2009; Hartmann et al., 2020; Zhang et al., 2022). First, individual and organizational resilience as well as team efficacy are important and interrelated determinants for employees in hospitals to adapt better with the COVID-19 pandemic. Organizational resilience seems to be a critical antecedent variable for individual resilience and team efficacy during the COVID-19 pandemic. Hereby, organizational resilience is not the sum of resilient employees, nor does it function independently of employees; rather, it relies on the interdependence of capacities at each level. In other words, resilient employees perceive their organization to be more resilient, and it seems to be easier for employees to be resilient in organizations with high organizational resilience. This is in line with research on promoting organizational resilience (in preparation for an adverse event), which in turn results in higher individual resilience (Teng-Calleja et al., 2020) and approaches to resilience that understand resilience as a reciprocal process involving employees and their organization (Kuntz et al., 2016).

Second, our study took a first step towards the empirically underexplored relationship between individual resilience and the demonstration of resilience by revealing the mediating roles of team efficacy and organizational resilience in this relationship. On the individual and organizational level of transformation, we found a partial mediation effect of organizational resilience on the relationship between individual resilience and perceived transformation. Team efficacy partially mediated the relationship between individual resilience and individual transformation as well as team transformation but not on its own for organizational transformation. In this case, organizational resilience was necessary in addition to team efficacy to partially mediate the relationship. Furthermore, people with high individual resilience are particularly likely to experience higher team efficacy and to perceive higher organizational resilience. Team efficacy relates positively to a sense of organizational resilience, which subsequentially will relate to positive perceptions of transformations. As there is no direct link between individual resilience and factors that demonstrate resilience at the team level, organizational resilience and team efficacy fully mediated the relationship between individual resilience and perceptions of team transformation. These results emphasize the need to consider the construct of resilience holistically and as a cross level construct (Zhang et al., 2022). Our results support the logical premise that organizational resilience enhances the capability to cope and learn within organizations at both the individual and the team level. This pattern of results points to benefits for healthcare workers and hospitals to boost resilience capacities.

Third, our conceptualization of resilience outcomes as positive perceptions of transformation is in line with the proposition that “resilient behaviors among employees will be related to positive outcomes, even when circumstances are challenging or highly stressful, but only to the extent that the organization fosters a resilience-building context” (Kuntz et al., 2016, p. 460). Our research extends this understanding by showing that resilience across levels is positively related to perceptions of transformation. Moreover, organizational resilience had the largest impact on perceptions of individual and organizational transformation. Hospitals with highly committed leaders, organizational awareness, good preparation, and flexibility as well as a just and learning culture were better able to adapt to the pandemic situation as a whole organization and for their members. This supports the importance of resources that allow for proactive coping strategies (job demands–resources theory, conservation of resources theory) and underlines that frontline workers experience positive changes such as posttraumatic growth during COVID-19 (Chen et al., 2021).

Fourth, our study reveals deeper insights into emergent phenomena at the collective level during a pandemic (response and adaptation phase; Ponomarov and Holcomb, 2009). Organizations have been described in resilience research as complex systems with interconnected agents forming a network of nonlinear interactions (Bhamra et al., 2011). These interactions inhibit or facilitate emergent phenomena such as organizational resilience and team efficacy. In general, efficacy beliefs at the individual and team level are important predictors of behavior (e.g., Sonnentag and Volmer, 2009). In times of crisis, they are still critical, but resilience mechanisms/capacities expand the resources needed to adapt and learn. Our data show that healthcare workers report high team efficacy, but organizational resilience must have emerged and must be facilitated to enable resilient behavior at the individual level. This indicates restrictions of social-cognitive approaches to resilience. Social-cognitive theory assumes that people have the power to control, transform, and develop their increasingly complex environments (Bandura, 2002). People therefore have the ability to adapt flexibly to the most diverse environments and to act proactively. In a pandemic, external forces (e.g., social distance, quarantine) restricted this proactive agency, making individual choices and behavior more dependent on higher level guidelines.

Our results are in line with research on the importance of organizational resilience and organizational support during the COVID-19 pandemicand supports the notion put forth by Rodríguez-Sánchez et al. (2021) regarding the human side of building organizational resilience and the need to integrate organizational factors to understand the complexities of team resilience. Hence it seems to be the case that the relevance of team and organizational levels changes in a crisis situation such as the COVID-19 pandemic. In crises, the organizational framework conditions become of utmost importance (Kreh et al., 2021; Zhang et al., 2022). Organizational practices (e.g., limiting change in task setting and team-related work) minimize the burnout of frontline workers (Sklar et al., 2021). Organizational justice (Kreh et al., 2021) and resilient focused leadership behavior (Giordano et al., 2022) increase the well-being of hospital staff. Building resilient healthcare systems is crucial to maintain high-quality healthcare even during a crisis (Haldane et al., 2021; Orru et al., 2021).

Finally, our results reveal the resilience of healthcare workers in hospitals in Germany (at least that of the healthcare workers in our sample; for limitations see below). We have summarized empirical results on how German healthcare providers and their employees have dealt with the crisis, closing a gap in the literature. Our results indicate that resilience indeed has been a highly relevant phenomenon for healthcare organizations to maintain their workforce during the pandemic. We have further extended the work on resilience in hospitals by following a holistic approach and by taking various occupation types into account.

### Limitations and avenues for future research

This study does not come without some limitations. Given the highly demanding nature of the situation in hospitals as well as in private lives during the pandemic, a convenience sample was used. Generalizations to healthcare providers and their employees can therefore not be derived. The statements and interpretations made here can therefore only be applied to the demographic groups that participated in the survey. As this is not a representative survey, we might have missed some stakeholders with specific backgrounds, for example, a migration background. In the health sector in Germany it is estimated that between 11 and 18% of the employees have a migration background/experience (Habermann and Stagge, 2015). We had pretested our questionnaire in hospitals (also before the pandemic) and did not account for language fluency. We speculate that people with migration experience (as a marginalized group) might face additional stressors during the pandemic. This also links to highly stressed groups that were not accessible to us because of their limited (time) capacities for answering an online survey. Participation in the online survey was optional; no benefits were offered. Therefore, we encourage future research to consider more nuanced approaches to meet the diversity of stakeholders.

Although our results support a strong interrelation between the individual, team, and organizational level, the use of cross-sectional data necessitated a correlational structure. Hence, this precludes making inferences of causality and does not allow us to investigate causal effects. Thus, we cannot disentangle whether organizational resilience is necessary as a framework for building individual resilience and team efficacy or whether individual and team efficacy are the “microfoundation of organisation-level resilience” (Hartmann et al., 2020). Future research is needed to investigate causal relationships between individual, team, and organizational resilience, for instance, by applying experimental research designs. Further, we only were able to collect data of individuals on their perceptions at different levels (individual resilience, team efficacy, organizational resilience) across different hospitals. Thus, we are not able to present team or organizational level differences in the concepts. Future research should consider collecting nested data on different levels of resilience to allow for multi-level analysis.

The cross-sectional design further does not allow for any assumptions regarding the development of resilience over time. The study was conducted in the third and fourth quarter of 2020, just after the first COVID-19 wave in Germany but long before the current state of the COVID-19 pandemic (nearly 2 years on, at this writing). Our results thus provide no insights into the subsequent waves of the COVID-19 pandemic. Many healthcare workers have resigned their jobs although our data showed (surprisingly) proactive and positive attitudes and perceptions. What role does time or duration of an adverse event play? What roles do preparation before and reflection after a crisis play? A deeper understanding of enhancing but also disempowering resilience processes across time is needed. Both longitudinal data and in-depth case studies are needed to be able to describe the processes of empowering and disempowering (e.g., *via* follow-up data collection after the pandemic) and to identify factors that foster persistence for resilient behavior at work.

Furthermore, comparative studies are needed to capture and embrace the dynamic character of resilience and its multiple potential pathways when dealing with a crisis within one hospital. Cross-sectional designs can reveal something as a ‘good practice’ but might miss the unique character of each hospital in dealing with a pandemic situation. In addition, we focused on one type of adverse event in one industry in one country (COVID-19 pandemic in healthcare providers in Germany). Hence, we cannot assume that our results are generalizable to other industries, other countries, other types of adverse events, or other phases of a crisis. Future research might investigate whether our findings can be replicated in other industries and other countries. To ensure comparability of results, it is recommended to use similar measures across studies. In cross-country comparisons, national characteristics of the healthcare systems might be another potential aspect that needs to be addressed.

To ensure reliability of the collected data, resilience at collective levels needs further clarification and translation in validated measures. Team resilience is conceptualized as a second-order emergent concept, whereas organizational resilience also follows emergent collective states but addresses more institutional processes. We decided to stick with a first-order concept such as team efficacy, while remaining aware of disregarding aspects of team resilience. Further research is needed to clarify the nature of the concepts and appropriate measurement approaches. One promising path would be to validate the short measures of organizational resilience we used in further studies. Such validated short scales could benefit research on occupations under high time pressure, as, for instance, the healthcare sector. Also, the development and validation of a reliable short scale measure for team resilience seems fruitful for future research.

Another limitation of our study is the use of self-report data. Although the use of self-reported data was appropriate for many of the variables we studied, a non-self-report measure of the outcomes in the hospital context would have been more ideal. We encourage future research to integrate organizational, team or individual performance measures to address this limitation. This also applies to our measure of perceptions of transformation, which can be interpreted as a cognitive measure. Future research on combinations of cognitive and behavioral measures would improve our picture of resilience and its demonstration.

Future research should also address the ‘dark side’ of resilience (Williams et al., 2017). Resilience might come at a cost (e.g., self-enhancing bias, positive illusions), which also could bias the (positive) answers in our sample. Enabling people to be energetic and happy might also inhibit learning and slow down responses to emerging threats (in Williams et al., 2017). Future research designs on resilience should integrate or be aware of this perspective.

### Implications

The results from a large online survey of German healthcare workers during the COVID-19 pandemic have some important theoretical and practical implications. The present study looked at the interplay of individual resilience characteristics and collective resilience in hospitals and their effects on transformation during the pandemic. Results indicate indeed that resilience is a highly interrelated construct on the individual, team, and organizational level. Both research and practical recommendations should thus conceptualize and derive measures to foster resilience on all three levels. Both practitioners and researchers can benefit from a more holistic approach because such frameworks account for interactions and complexities between variables at different levels and in doing so direct attention to important areas where interventions can build resilience within healthcare providers.

This study highlights further that during a crisis, organizational capabilities are of utmost importance. Whereas team efficacy is crucial for performance in regular work operations, this shifts to the organizational level during a pandemic. Organizational processes must be created to maintain and promote resilient behavior of employees and teams. Organizations that are flexible in adjusting work processes should consider aspects of team efficacy and support resilient behavior in teams. For example, monitoring aspects of resilience might prevent physician burnout and reduced workforce capacities (Darrow and Eseonu, 2017). Also, continuous assessment within organizations on the multiple levels of resilience is recommended to detect potential needs within an organization. As such, evaluations conducted during normal operations (i.e., noncrisis times) can also serve as a benchmark tool to examine developments within an organization over time or after specific companywide trainings.

Individual resilience can be strengthened by long-term-oriented resilience training programs, which, for example, positively affect job satisfaction (Liossis et al., 2009; Vanhove et al., 2016). Vanhove et al. (2016) showed in their meta-analysis that resilience-building programs (as well as other prevention programs) in organizations have a modest effect across health and performance criteria, but those effects diminish over time. Their explanation was that learned skills were not being used. Consequently, fostering resilience is a continuous process that should be aligned across levels by human resources departments, as proposed by Branicki et al. (2019). Nevertheless, there is a lack of studies on holistic resilience-building programs. It hence seems fruitful to develop programs at different levels in hospitals to foster resilience holistically across levels and in addition to evaluate their effectiveness.

## Conclusion

In this study we shed light on the subjective experiences of employees in hospitals (healthcare workers, physicians, administrative staff) during the first wave of the COVID-19 pandemic in Germany. Our goal was to gain deeper insights into the interrelations of different levels of resilience in hospitals. In order to better understand the determinants, underlying mechanisms and consequences of resilience, we were especially interested in the interconnections of organizational and individual resilience and their relation to team efficacy as well as in the questions, how the change caused by COVID-19 is perceived at different levels in hospitals.

Our results reveal that organizational resilience becomes of utmost importance in a pandemic, and, when in place, enables both resilient behavior of employees and team-efficacy. Thus, organizational resilience enhances the capability to cope and learn within organizations at both the individual and the team level in hospitals during the pandemic. Moreover, resilience leads to positive perceptions of transformation (caused by COVID-19 pandemic) at different levels in hospitals, when employees experienced support by their organization and when they are able to believe in the competencies of their teams.

Our results indicate that resilience indeed has been a highly relevant phenomenon for healthcare organizations to maintain their workforce during the pandemic. Collective phenomena such as team efficacy and even more organizational resilience function as a catalyst during a pandemic. Thus, healthcare providers should conceptualize and derive measures to foster resilience especially on the organizational level, but also of their employees and teams.

Further research is needed to gain deeper insights into the multi-level structure of resilience and to integrate a multimodal and interdisciplinary perspective (e.g., socioecological) to foster resilience for healthcare providers during and after COVID-19. Further considerations should be taken regarding the ‘dark side’ of resilience.

## Data availability statement

The raw data supporting the conclusions of this article will be made available by the authors, without undue reservation.

## Ethics statement

Ethical review and approval was not required for the study on human participants in accordance with the local legislation and institutional requirements. The participants provided their written informed consent to participate in this study.

## Author contributions

DG, NM, and JW contributed to the conception and design of the study. NM and EH organized the database and performed the statistical analysis. DG and EH wrote the first draft of the manuscript. All authors contributed to the manuscript revision and read and approved the submitted version.

## Funding

This work was supported by the German Federal Ministry of Education and Research (BMBF) and Zwanzig20—InfectControl—EKOS under grant number 03ZZ0817D.

## Conflict of interest

The authors declare that the research was conducted in the absence of any commercial or financial relationships that could be construed as a potential conflict of interest.

## Publisher’s note

All claims expressed in this article are solely those of the authors and do not necessarily represent those of their affiliated organizations, or those of the publisher, the editors and the reviewers. Any product that may be evaluated in this article, or claim that may be made by its manufacturer, is not guaranteed or endorsed by the publisher.

## Footnotes

^1^The analyzed data set is part of a larger research project. Not all collected variables are reported in this paper.

## References

[ref1] Aburn G. Gott M. Hoare K. (2016). What is resilience? An integrative review of the empirical literature. J. Adv. Nurs. 72, 980–1000. doi: 10.1111/jan.12888, PMID: 26748456

[ref2] Alliger G. M. Cerasoli C. P. Tannenbaum S. I. Vessey W. B. (2015). Team resilience: how teams flourish under pressure. Organ. Dyn. 44, 176–184. doi: 10.1016/j.orgdyn.2015.05.003

[ref3] Avanzi L. Schuh S. C. Fraccaroli F. van Dick R. (2015). Why does organizational identification relate to reduced employee burnout? The mediating influence of social support and collective efficacy. Work Stress 29, 1–10. doi: 10.1080/02678373.2015.1004225

[ref4] Bandura A. (2000). Exercise of human agency through collective efficacy. Curr. Dir. Psychol. Sci. 9, 75–78. doi: 10.1111/1467-8721.00064

[ref5] Bandura A. (2002). Social cognitive theory in cultural context. Appl. Psychol. 51, 269–290. doi: 10.1111/1464-0597.00092

[ref6] Bandyopadhyay S. Baticulon R. E. Kadhum M. Alser M. Ojuka D. K. Badereddin Y.. (2020). Infection and mortality of healthcare workers worldwide from COVID-19: a systematic review. BMJ Glob. Health 5, e003097. doi: 10.1136/bmjgh-2020-003097, PMID: 33277297 PMC7722361

[ref7] Banerjee D. Sathyanarayana Rao T. S. Kallivayalil R. A. Javed A. (2021). Psychosocial framework of resilience: navigating needs and adversities during the pandemic, a qualitative exploration in the Indian frontline physicians. Front. Psychol. 12:622132. doi: 10.3389/fpsyg.2021.622132, PMID: 33796046 PMC8007982

[ref8] Barasa E. Mbau R. Gilson L. (2018). What is resilience and how can it be nurtured? A systematic review of empirical literature on organizational resilience. Int. J. Health Policy Manag. 7, 491–503. doi: 10.15171/ijhpm.2018.06, PMID: 29935126 PMC6015506

[ref9] Benfante A. Di Tella M. Romeo A. Castelli L. (2020). Traumatic stress in healthcare workers during COVID-19 pandemic: a review of the immediate impact. Front. Psychol. 11:569935. doi: 10.3389/fpsyg.2020.569935, PMID: 33192854 PMC7645025

[ref10] Bhamra R. Dani S. Burnard K. (2011). Resilience: the concept, a literature review and future directions. Int. J. Prod. Res. 49, 5375–5393. doi: 10.1080/00207543.2011.563826

[ref11] Bowers C. Kreutzer C. Cannon-Bowers J. Lamb J. (2017). Team resilience as a second-order emergent state: a theoretical model and research directions. Front. Psychol. 8:1360. doi: 10.3389/fpsyg.2017.01360, PMID: 28861013 PMC5562719

[ref12] Bozdağ F. Ergün N. (2021). Psychological resilience of healthcare professionals during COVID-19 pandemic. Psychol. Rep. 124, 2567–2586. doi: 10.1177/0033294120965477, PMID: 33050800 PMC7557235

[ref13] Branicki L. Steyer V. Sullivan-Taylor B. (2019). Why resilience managers aren’t resilient, and what human resource management can do about it. Int. J. Hum. Resource Manag. 30, 1261–1286. doi: 10.1080/09585192.2016.1244104

[ref14] Britt T. W. Shen W. Sinclair R. R. Grossman M. R. Klieger D. M. (2016). How much do we really know about employee resilience? Ind. Organ. Psychol. 9, 378–404. doi: 10.1017/iop.2015.107

[ref15] Capone V. Borrelli R. Marino L. Schettino G. (2022). Mental well-being and job satisfaction of hospital physicians during COVID-19: relationships with efficacy beliefs, organizational support, and organizational non-technical skills. Int. J. Environ. Res. Public Health 19, 3734. doi: 10.3390/ijerph19063734, PMID: 35329420 PMC8948767

[ref16] Carmeli A. Gelbard R. Reiter-Palmon R. (2013). Leadership, creative problem-solving capacity, and creative performance: the importance of knowledge sharing. Hum. Resource Manag. 52, 95–121. doi: 10.1002/hrm.21514

[ref17] Chen R. Sun C. Chen J.-J. Jen H.-J. Kang X. L. Kao C.-C.. (2021). A large-scale survey on trauma, burnout, and posttraumatic growth among nurses during the COVID-19 pandemic. Int. J. Ment. Health Nurs. 30, 102–116. doi: 10.1111/inm.12796, PMID: 33107677 PMC7894338

[ref18] Chen G. Thomas B. Wallace J. C. (2005). A multilevel examination of the relationships among training outcomes, mediating regulatory processes, and adaptive performance. J. Appl. Psychol. 90, 827–841. doi: 10.1037/0021-9010.90.5.827, PMID: 16162057

[ref19] Cohen S. Nica E. (2021). COVID-19 pandemic-related emotional anxiety, perceived risk of infection, and acute depression among primary care providers. Psychosoc. Issues Hum. Resour. Manag. 9, 7–20. doi: 10.22381/pihrm9220211

[ref20] Couarraze S. Delamarre L. Marhar F. Quach B. Jiao J. Avilés Dorlhiac R.. (2021). The major worldwide stress of healthcare professionals during the first wave of the COVID-19 pandemic: the international COVISTRESS survey. PLoS One 16:e0257840. doi: 10.1371/journal.pone.0257840, PMID: 34614016 PMC8494302

[ref21] Coulombe S. Pacheco T. Cox E. Khalil C. Doucerain M. M. Auger E.. (2020). Risk and resilience factors during the COVID-19 pandemic: a snapshot of the experiences of Canadian workers early on in the crisis. Front. Psychol. 11:580702. doi: 10.3389/fpsyg.2020.580702, PMID: 33343455 PMC7744587

[ref22] Coutu D. L. (2002). How resilience works. Harvard Bus. Rev. 80, 46–56. 12024758

[ref23] Darrow L. Eseonu C. I. (2017). “Development of a resilience analysis grid survey tool for healthcare,” in Proceedings of the 2017 Industrial and Systems Engineering Conference, eds. Coperich K. Nembhard H. B. Cudney E. (Pittsburgh, PA: Institute of Industrial and Systems Engineers (IISE)), 1163–1168.

[ref24] Day A. L. Sibley A. Scott N. Tallon J. M. Ackroyd-Stolarz S. (2009). Workplace risks and stressors as predictors of burnout: the moderating impact of job control and team efficacy. Can. J. Admin. Sci. 26, 7–22. doi: 10.1002/cjas.91

[ref25] de Clercq D. Belausteguigoitia I. (2017). Mitigating the negative effect of perceived organizational politics on organizational citizenship behavior: moderating roles of contextual and personal resources. J. Manage. Organ. 23, 689–708. doi: 10.1017/jmo.2017.7

[ref26] de Terte I. Stephens C. (2014). Psychological resilience of workers in high-risk occupations. Stress. Health 30, 353–355. doi: 10.1002/smi.2627, PMID: 25476960

[ref27] Di Trani M. Mariani R. Ferri R. de Berardinis D. Frigo M. G. (2021). From resilience to burnout in healthcare workers during the COVID-19 emergency: the role of the ability to tolerate uncertainty. Front. Psychol. 12:646435. doi: 10.3389/fpsyg.2021.646435, PMID: 33935905 PMC8085585

[ref28] Douillet D. Caillaud A. Riou J. Miroux P. Thibaud E. Noizet M.. (2021). Assessment of physicians' resilience level during the COVID-19 pandemic. Transl. Psychiatry 11, 283. doi: 10.1038/s41398-021-01395-7, PMID: 33980816 PMC8114969

[ref29] Driskell J. E. Salas E. (1991). Group decision making under stress. J. Appl. Psychol. 76, 473–478. doi: 10.1037/0021-9010.76.3.473

[ref30] Dryhurst S. Schneider C. R. Kerr J. Freeman A. L. J. Recchia G. van der Bles A. M.. (2020). Risk perceptions of COVID-19 around the world. J. Risk Res. 23, 994–1006. doi: 10.1080/13669877.2020.1758193

[ref31] Duchek S. (2020). Organizational resilience: a capability-based conceptualization. Bus. Res. 13, 215–246. doi: 10.1007/s40685-019-0085-7

[ref32] Duffy J. R. Culp S. Padrutt T. (2018). Description and factors associated with missed nursing care in an acute care community hospital. J. Nurs. Admin. 48, 361–367. doi: 10.1097/NNA.0000000000000630 30001260

[ref33] Fino E. Bonfrate I. Fino V. Bocus P. Russo P. M. Mazzetti M. (2021). Harnessing distress to boost growth in frontline healthcare workers during COVID-19 pandemic: the protective role of resilience, emotion regulation and social support. Psychol. Med. 1–3, 519. doi: 10.1017/S0033291721000519, PMID: 33565390 PMC7900668

[ref35] Flint-Taylor J. Cooper C. L. (2017). “Team resilience: shaping up for the challenges ahead” in Managing for Resilience: A Practical guide to Individual Wellbeing and Organizational Performance. ed. Crane M. F. (London, New York: Routledge), 129–149. doi: 10.4324/9781315648033-9

[ref36] Gichuhi J. M. (2021). Shared leadership and organizational resilience: a systematic literature review. Int. J. Org. Lead. 10, 67–88. doi: 10.33844/ijol.2021.60536

[ref37] Giordano F. Cipolla A. Ungar M. (2022). Building resilience for healthcare professionals working in an Italian red zone during the COVID-19 outbreak: a pilot study. Stress. Health 38, 234–248. doi: 10.1002/smi.3085, PMID: 34312986 PMC9292917

[ref38] Gladstein D. L. Reilly N. P. (1985). Group decision making under threat: the tycoon game. Acad. Manag. J. 28, 613–627. doi: 10.2307/256117

[ref39] Gonçalves L. Sala R. Navarro J.-B. (2022). Resilience and occupational health of healthcare workers: a moderator analysis of organizational resilience and sociodemographic attributes. Int. Arch. Occup. Environ. Health 95, 223–232. doi: 10.1007/s00420-021-01725-8, PMID: 34076733 PMC8170862

[ref40] Gucciardi D. F. Crane M. Ntoumanis N. Parker S. K. Thøgersen-Ntoumani C. Ducker K. J.. (2018). The emergence of team resilience: a multilevel conceptual model of facilitating factors. J. Occup. Organ. Psychol. 91, 729–768. doi: 10.1111/joop.12237

[ref41] Gully S. M. Incalcaterra K. A. Joshi A. Beaubien J. M. (2002). A meta-analysis of team-efficacy, potency, and performance: interdependence and level of analysis as moderators of observed relationships. J. Appl. Psychol. 87, 819–832. doi: 10.1037/0021-9010.87.5.819, PMID: 12395807

[ref42] Habermann M. Stagge M. (2015). “Menschen mit Migrationshintergrund in der professionellen Pflege” in Zukunft der Pflege. ed. Zängl P. (Wiesbaden, Germany: Springer), 159–175.

[ref43] Haldane V. de Foo C. Abdalla S. M. Jung A.-S. Tan M. Wu S.. (2021). Health systems resilience in managing the COVID-19 pandemic: lessons from 28 countries. Nat. Med. 27, 964–980. doi: 10.1038/s41591-021-01381-y, PMID: 34002090

[ref44] Hartmann S. Weiss M. Newman A. Hoegl M. (2020). Resilience in the workplace: a multilevel review and synthesis. Appl. Psychol. 69, 913–959. doi: 10.1111/apps.12191

[ref45] Hartwig A. Clarke S. Johnson S. Willis S. (2020). Workplace team resilience: a systematic review and conceptual development. Organ. Psychol. Rev. 10, 169–200. doi: 10.1177/2041386620919476

[ref46] Hayes A. F. (2017). Introduction to Mediation, Moderation, and Conditional Process Analysis: A Regression-Based Approach. New York: The Guilford Press.

[ref47] Heckemann B. Hahn S. Halfens R. J. G. Richter D. Schols J. M. G. A. (2019). Patient and visitor aggression in healthcare: a survey exploring organisational safety culture and team efficacy. J. Nurs. Manage. 27, 1039–1046. doi: 10.1111/jonm.12772, PMID: 30888740

[ref48] Hillmann J. Guenther E. (2021). Organizational resilience: a valuable construct for management research? Int. J. Manage. Rev. 23, 7–44. doi: 10.1111/ijmr.12239

[ref49] Horne J. F. (1997). The coming age of organizational resilience. Bus. Forum 22, 24–29.

[ref50] Horne J. F. Orr J. E. (1998). Assessing behaviors that create resilient organizations. Employ. Relat. Today 24, 29–39.

[ref51] Huffman E. M. Athanasiadis D. I. Anton N. E. Haskett L. A. Doster D. L. Stefanidis D.. (2021). How resilient is your team? Exploring healthcare providers’ well-being during the COVID-19 pandemic. Am. J. Surg. 221, 277–284. doi: 10.1016/j.amjsurg.2020.09.005, PMID: 32994041 PMC7486626

[ref52] Jalloh M. F. Sengeh P. Monasch R. Jalloh M. B. DeLuca N. Dyson M.. (2017). National survey of Ebola-related knowledge, attitudes and practices before the outbreak peak in Sierra Leone: august 2014. BMJ Glob. Health 2:e000285. doi: 10.1136/bmjgh-2017-000285, PMID: 29259820 PMC5728302

[ref53] Jeffcott S. A. Ibrahim J. Cameron P. (2009). Resilience in healthcare and clinical handover. Qual. Saf. Health Care 18, 256–260. doi: 10.1136/qshc.2008.030163, PMID: 19651927

[ref54] Jex S. M. Bliese P. D. (1999). Efficacy beliefs as a moderator of the impact of work-related stressors: a multilevel study. J. Appl. Psychol. 84, 349–361. doi: 10.1037/0021-9010.84.3.349, PMID: 10380416

[ref55] Jo S. Kurt S. Bennett J. A. Mayer K. Pituch K. A. Simpson V.. (2021). Nurses’ resilience in the face of coronavirus (COVID-19): an international view. Nurs. Health Sci. 23, 646–657. doi: 10.1111/nhs.12863, PMID: 34169629 PMC8447204

[ref56] Kärner T. Bottling M. Friederichs E. Sembill D. (2021). Between adaptation and resistance: a study on resilience competencies, stress, and well-being in German VET teachers. Front. Psychol. 12:619912. doi: 10.3389/fpsyg.2021.619912, PMID: 34295278 PMC8289907

[ref57] Khalid I. Khalid T. J. Qabajah M. R. Barnard A. G. Qushmaq I. A. (2016). Healthcare workers emotions, perceived stressors and coping strategies during a MERS-CoV outbreak. Clin. Med. Res. 14, 7–14. doi: 10.3121/cmr.2016.1303, PMID: 26847480 PMC4851451

[ref58] Koh D. Lim M. K. Chia S. E. Ko S. M. Qian F. Ng V.. (2005). Risk perception and impact of severe acute respiratory syndrome (SARS) on work and personal lives of healthcare workers in Singapore: what can we learn? Med. Care 43, 676–682. doi: 10.1097/01.mlr.0000167181.36730.cc, PMID: 15970782

[ref60] Kreh A. Brancaleoni R. Magalini S. C. Chieffo D. P. R. Flad B. Ellebrecht N.. (2021). Ethical and psychosocial considerations for hospital personnel in the Covid-19 crisis: moral injury and resilience. PLoS One 16:e0249609. doi: 10.1371/journal.pone.0249609, PMID: 33798251 PMC8018614

[ref61] Kuntz J. R. C. Näswall K. Malinen S. (2016). Resilient employees in resilient organizations: flourishing beyond adversity. Ind. Organ. Psychol. 9, 456–462. doi: 10.1017/iop.2016.39

[ref62] Labrague L. J. (2021). Psychological resilience, coping behaviours and social support among healthcare workers during the COVID-19 pandemic: a systematic review of quantitative studies. J. Nurs. Manage. 29, 1893–1905. doi: 10.1111/jonm.13336, PMID: 33843087 PMC8250179

[ref63] Labrague L. J. de los Santos J. A. A. (2020). COVID-19 anxiety among front-line nurses: predictive role of organisational support, personal resilience and social support. J. Nurs. Manage. 28, 1653–1661. doi: 10.1111/jonm.13121, PMID: 32770780 PMC7436313

[ref64] Lai X. Wang X. Yang Q. Xu X. Tang Y. Liu C.. (2020). Will healthcare workers improve infection prevention and control behaviors as COVID-19 risk emerges and increases, in China? Resist. Infect. Control 9, 83–89. doi: 10.1186/s13756-020-00746-1, PMID: 32527300 PMC7289224

[ref65] Larson M. Luthans F. (2006). Potential added value of psychological capital in predicting work attitudes. J. Leadersh. Organ. Stud. 13, 75–92. doi: 10.1177/10717919070130020601

[ref66] Lee T. W. Ko Y. K. (2010). Effects of self-efficacy, affectivity and collective efficacy on nursing performance of hospital nurses. J. Adv. Nurs. 66, 839–848. doi: 10.1111/j.1365-2648.2009.05244.x, PMID: 20423371

[ref67] Lengnick-Hall C. A. Beck T. E. Lengnick-Hall M. L. (2011). Developing a capacity for organizational resilience through strategic human resource management. Hum. Res. Manage. Rev. 21, 243–255. doi: 10.1016/j.hrmr.2010.07.001

[ref68] Lindsley D. H. Brass D. J. Thomas J. B. (1995). Efficacy-performing spirals: a multilevel perspective. Acad. Manag. Rev. 20, 645–678. doi: 10.5465/amr.1995.9508080333

[ref69] Linnenluecke M. K. (2017). Resilience in business and management research: a review of influential publications and a research agenda. Int. J. Manage. Rev. 19, 4–30. doi: 10.1111/ijmr.12076

[ref70] Liossis P. L. Shochet I. M. Millear P. M. Biggs H. (2009). The promoting adult resilience (PAR) program: the effectiveness of the second, shorter pilot of a workplace prevention program. Behav. Change 26, 97–112. doi: 10.1375/bech.26.2.97

[ref71] Luthans F. Youssef C. M. (2007). Emerging positive organizational behavior. J. Manage. 33, 321–349. doi: 10.1177/0149206307300814

[ref72] Mallak L. A. (1998). Measuring resilience in healthcare provider organizations. Health Manpow. Manag. 24, 148–152. doi: 10.1108/09552069810215755, PMID: 10346317

[ref73] Mallak L. A. Yildiz M. (2016). Developing a workplace resilience instrument. Work 54, 241–253. doi: 10.3233/WOR-162297, PMID: 27259179

[ref74] Martin-Breen P. Anderies J. M. (2011). Resilience: a literature review. New York: Rockefeller Foundation.

[ref75] Martinez-Corts I. Demerouti E. Bakker A. B. Boz M. (2015). Spillover of interpersonal conflicts from work into nonwork: a daily diary study. J. Occup. Health Psychol. 20, 326–337. doi: 10.1037/a0038661, PMID: 25602278

[ref76] Masten A. S. (2001). Ordinary magic: resilience processes in development. Am. Psychol. 56, 227–238. doi: 10.1037//0003-066X.56.3.227, PMID: 11315249

[ref77] Matheson C. Robertson H. D. Elliott A. M. Iversen L. Murchie P. (2016). Resilience of primary healthcare professionals working in challenging environments: a focus group study. Br. J. Gen. Pract. 66, e507–e515. doi: 10.3399/bjgp16X685285, PMID: 27162205 PMC4917054

[ref78] McCray J. Palmer A. Chmiel N. (2016). Building resilience in health and social care teams. Personnel Rev. 45, 1132–1155. doi: 10.1108/PR-04-2014-0095

[ref79] McEwen K. Boyd C. M. (2018). A measure of team resilience: developing the resilience at work team scale. J. Occup. Environ. Med. 60, 258–272. doi: 10.1097/JOM.0000000000001223 29112630

[ref80] Mhango M. Dzobo M. Chitungo I. Dzinamarira T. (2020). COVID-19 risk factors among health workers: a rapid review. Safe. Health Work 11, 262–265. doi: 10.1016/j.shaw.2020.06.001, PMID: 32995051 PMC7502606

[ref81] Mitchell K. Lăzăroiu G. (2021). Depressive symptoms, emotional exhaustion, and psychological trauma symptoms in frontline healthcare workers during the COVID-19 outbreak. Psychosociol. Issues Hum. Resour. Manag. 9, 119–132. doi: 10.22381/pihrm9220219

[ref82] Morgan P. B. C. Fletcher D. Sarkar M. (2017). Recent developments in team resilience research in elite sport. Curr. Opin. Psychol. 16, 159–164. doi: 10.1016/j.copsyc.2017.05.013, PMID: 28813342

[ref83] Morgan P. B. Fletcher D. Sarkar M. (2019). Developing team resilience: a season-long study of psychosocial enablers and strategies in a high-level sports team. Psychol. Sport Exerc. 45:101543. doi: 10.1016/j.psychsport.2019.101543

[ref84] Nemțeanu M.-S. Dinu V. Pop R.-A. Dabija D.-C. (2022). Predicting job satisfaction and work engagement behavior in the COVID-19 pandemic: a conservation of resources theory approach. E+M 25, 23–40. doi: 10.15240/tul/001/2022-2-002

[ref85] Nguyen L. H. Drew D. A. Graham M. S. Joshi A. D. Guo C.-G. Ma W.. (2020). Risk of COVID-19 among front-line health-care workers and the general community: a prospective cohort study. Lancet Pub. Health 5, e475–e483. doi: 10.1016/S2468-2667(20)30164-X, PMID: 32745512 PMC7491202

[ref86] Nielsen K. Yarker J. Randall R. Munir F. (2009). The mediating effects of team and self-efficacy on the relationship between transformational leadership, and job satisfaction and psychological well-being in healthcare professionals: a cross-sectional questionnaire survey. Int. J. Nurs. Stud. 46, 1236–1244. doi: 10.1016/j.ijnurstu.2009.03.001, PMID: 19345946

[ref87] Organizational Resilience Health Check (2019). Organizational Resilience HealthCheck. Available at: https://www.organisationalresilience.gov.au/HealthCheck/overview (Accessed February 07, 2019).

[ref88] Orru K. Nero K. Naevestad T.-O. Schieffelers A. Olson A. Airola M.. (2021). Resilience in care organisations: challenges in maintaining support for vulnerable people in Europe during the Covid-19 pandemic. Disasters 45, S48–S75. doi: 10.1111/disa.12526, PMID: 34874082 PMC9300196

[ref89] Pal R. Torstensson H. Mattila H. (2014). Antecedents of organizational resilience in economic crises: an empirical study of Swedish textile and clothing SMEs. Int. J. Product. Econ. 147, 410–428. doi: 10.1016/j.ijpe.2013.02.031

[ref90] Pangallo A. Zibarras L. Lewis R. Flaxman P. (2015). Resilience through the lens of interactionism: a systematic review. Psychol. Assess. 27, 1–20. doi: 10.1037/pas0000024, PMID: 25222438

[ref91] Podsakoff P. M. MacKenzie S. B. Lee J.-Y. Podsakoff N. P. (2003). Common method biases in behavioral research: a critical review of the literature and recommended remedies. J. Appl. Psychol. 88, 879–903. doi: 10.1037/0021-9010.88.5.879, PMID: 14516251

[ref92] Ponomarov S. Y. Holcomb M. C. (2009). Understanding the concept of supply chain resilience. Int. J. Logistics Manage. 20, 124–143. doi: 10.1108/09574090910954873

[ref93] Prayag G. Spector S. Orchiston C. Chowdhury M. (2020). Psychological resilience, organizational resilience and life satisfaction in tourism firms: insights from the Canterbury earthquakes. Curr. Iss. Tourism 23, 1216–1233. doi: 10.1080/13683500.2019.1607832

[ref94] Raetze S. Duchek S. Maynard M. T. Kirkman B. L. (2021). Resilience in organizations: an integrative multilevel review and editorial introduction. Group Organ. Manage. 46, 607–656. doi: 10.1177/10596011211032129

[ref95] Richtnér A. Löfsten H. (2014). Managing in turbulence: how the capacity for resilience influences creativity. R&D Manage. 44, 137–151. doi: 10.1111/radm.12050

[ref96] Rieckert A. Schuit E. Bleijenberg N., Cate, D. ten, Lange, W. de, de Man-van Ginkel J. M.. (2021). How can we build and maintain the resilience of our healthcare professionals during COVID-19? Recommendations based on a scoping review. BMJ Open 11:e043718. doi: 10.1136/bmjopen-2020-043718, PMID: 33408212 PMC7789206

[ref97] Riguzzi M. Gashi S. (2021). Lessons from the first wave of COVID-19: work-related consequences, clinical knowledge, emotional distress, and safety-conscious behavior in healthcare workers in Switzerland. Front. Psychol. 12:628033. doi: 10.3389/fpsyg.2021.628033, PMID: 33633652 PMC7899962

[ref98] Riolli L. Savicki V. (2003). Information system organizational resilience. Omega 31, 227–233. doi: 10.1016/S0305-0483(03)00023-9

[ref99] Robertson H. D. Elliott A. M. Burton C. Iversen L. Murchie P. Porteous T.. (2016). Resilience of primary healthcare professionals: a systematic review. Br. J. Gen. Pract. 66, e423–e433. doi: 10.3399/bjgp16x685261, PMID: 27162208 PMC4871308

[ref100] Rodríguez-Sánchez A. Guinot J. Chiva R. López-Cabrales Á. (2021). How to emerge stronger: antecedents and consequences of organizational resilience. J. Manage. Organ. 27, 442–459. doi: 10.1017/jmo.2019.5

[ref101] Sayegh L. Anthony W. P. Perrewé P. L. (2004). Managerial decision-making under crisis: the role of emotion in an intuitive decision process. Hum. Res. Manage. Rev. 14, 179–199. doi: 10.1016/j.hrmr.2004.05.002

[ref102] Sharma S. Sharma S. K. (2016). Team resilience: scale development and validation. Vision J. Bus. Perspect. 20, 37–53. doi: 10.1177/0972262916628952

[ref103] Shaukat N. Ali D. M. Razzak J. (2020). Physical and mental health impacts of COVID-19 on healthcare workers: a scoping review. Int. J. Emerg. Med. 13, 40–48. doi: 10.1186/s12245-020-00299-5, PMID: 32689925 PMC7370263

[ref104] Shin J. Taylor M. S. Seo M.-G. (2012). Resources for change: the relationships of organizational inducements and psychological resilience to employees' attitudes and behaviors toward organizational change. Acad. Manag. J. 55, 727–748. doi: 10.5465/amj.2010.0325

[ref105] Sklar M. Ehrhart M. G. Aarons G. A. (2021). COVID-related work changes, burnout, and turnover intentions in mental health providers: a moderated mediation analysis. Psychiatr. Rehabil. J. 44, 219–228. doi: 10.1037/prj0000480, PMID: 33998824 PMC8675296

[ref106] Smith J. G. Morin K. H. Wallace L. E. Lake E. T. (2018). Association of the nurse work environment, collective efficacy, and missed care. West. J. Nurs. Res. 40, 779–798. doi: 10.1177/0193945917734159, PMID: 28978300 PMC5878972

[ref107] Sonnentag S. Volmer J. (2009). Individual-level predictors of task-related teamwork processes. Group Organ. Manage. 34, 37–66. doi: 10.1177/1059601108329377

[ref108] Soucek R. Pauls N. Ziegler M., and & Schlett C. (2015). Entwicklung eines Fragebogens zur Erfassung resilienten Verhaltens bei der Arbeit. Wirtschaftspsychologie 17, 13–23.

[ref109] Soucek R. Ziegler M. Schlett C. Pauls N. (2016). Resilienz im Arbeitsleben: Eine inhaltliche Differenzierung von Resilienz auf den Ebenen von Individuen, Teams und Organisationen. Gruppe. Interaktion. Organ. 47, 131–137. doi: 10.1007/s11612-016-0314-x

[ref110] Sutcliffe K. M. Vogus T. J. (2003). “Organizing for resilience,” in Positive Organizational Scholarship: Foundations of a New Discipline, eds. Cameron K. S. Dutton J. E. Quinn R. E. (San Francisco, CA: Berrett-Koehler), 94–110.

[ref111] Tam C. C. Sun S. Yang X. Li X. Zhou Y. Shen Z. (2021). Psychological distress among HIV healthcare providers during the COVID-19 pandemic in China: mediating roles of institutional support and resilience. AIDS Behav. 25, 9–17. doi: 10.1007/s10461-020-03068-w, PMID: 33089356 PMC7577363

[ref112] Teng-Calleja M. Hechanova M. R. M. Sabile P. R. Villasanta A. P. V. P. (2020). Building organization and employee resilience in disaster contexts. Int. J. Workp. Health Manage. 13, 393–411. doi: 10.1108/IJWHM-09-2019-0122

[ref113] Toner E. S. McGinty M. Schoch-Spana M. Rose D. A. Watson M. Echols E.. (2017). A community checklist for health sector resilience informed by hurricane sandy. Health Secur. 15, 53–69. doi: 10.1089/hs.2016.0079, PMID: 28192055 PMC5551499

[ref114] Traylor A. M. Tannenbaum S. I. Thomas E. J. Salas E. (2021). Helping healthcare teams save lives during COVID-19: insights and countermeasures from team science. Am. Psychol. 76, 1–13. doi: 10.1037/amp0000750, PMID: 33119329 PMC8543842

[ref115] Vagni M. Maiorano T. Giostra V. Pajardi D. (2020). Coping with COVID-19: emergency stress, secondary trauma and self-efficacy in healthcare and emergency workers in Italy. Front. Psychol. 11:566912. doi: 10.3389/fpsyg.2020.566912, PMID: 33013603 PMC7494735

[ref116] Vanhove A. J. Herian M. N. Perez A. L. U. Harms P. D. Lester P. B. (2016). Can resilience be developed at work? A meta-analytic review of resilience-building programme effectiveness. J. Occup. Organ. Psychol. 89, 278–307. doi: 10.1111/joop.12123

[ref117] Vogus T. J. Sutcliffe K. M. (2007). “Organizational resilience: towards a theory and research agenda,” in 2007 IEEE International Conference on Systems, Man and Cybernetics, 3418–3422.

[ref118] Wachs P. Saurin T. A. Righi A. W. Wears R. L. (2016). Resilience skills as emergent phenomena: a study of emergency departments in Brazil and the United States. Appl. Ergon. 56, 227–237. doi: 10.1016/j.apergo.2016.02.012, PMID: 26972019

[ref119] Werner E. E. Smith R. S. (1979). An epidemiologic perspective on some antecedents and consequences of childhood mental health problems and learning disabilities: a report from the Kauai longitudinal study. J. Am. Acad. Child Psychiatry 18, 292–306. doi: 10.1016/S0002-7138(09)61044-X, PMID: 447961

[ref120] Williams T. A. Gruber D. A. Sutcliffe K. M. Shepherd D. A. Zhao E. Y. (2017). Organizational response to adversity: fusing crisis management and resilience research streams. Acad. Manage. Ann. 11, 733–769. doi: 10.5465/annals.2015.0134

[ref121] Wong T. Y. Koh G. C. H. Cheong S. K. Sundram M. Koh K. Chia S. E.. (2008). A cross-sectional study of primary-care physicians in Singapore on their concerns and preparedness for an avian influenza outbreak. Ann. Acad. Med. Singap. 37, 458–464. 18618056

[ref122] Wreathall J. (2006). “Properties of resilient organizations: an initial view,” in Resilience Engineering: Concepts and Precepts, eds. Woods D. D. Hollnagel E. Leveson N. (Aldershot, UK: Ashgate), 275–285.

[ref123] Youssef C. M. Luthans F. (2007). Positive organizational behavior in the workplace. J. Manage. 33, 774–800. doi: 10.1177/0149206307305562

[ref124] Zellars K. L. Perrewe P. L. Hochwarter W. A. (1999). Mitigating burnout among high-NA employees in healthcare: what can organizations do? J. Appl. Soc. Psychol. 29, 2250–2271. doi: 10.1111/j.1559-1816.1999.tb00109.x

[ref125] Zhang N. Yang S. Jia P. (2022). Cultivating resilience During the COVID-19 pandemic: a socioecological perspective. Annu. Rev. Psychol. 73, 575–598. doi: 10.1146/annurev-psych-030221-031857, PMID: 34579547

[ref126] Zhao Y. Ma D. Wan Z. Sun D. Li H. Sun J. (2020). Associations between work environment and implicit rationing of nursing care: a systematic review. J. Nurs. Manage. 28, 1841–1850. doi: 10.1111/jonm.12895, PMID: 31680364

