# Supplementary material for: Individual and organizational resilience—Insights from healthcare providers in Germany during the COVID-19 pandemic
Source: Front Psychol. 2022 Aug 25;13:965380. doi: 10.3389/fpsyg.2022.965380 (PMC9453859; doi:10.3389/fpsyg.2022.965380)
Supplement: Supplementary file 1 [file Data_Sheet_1.pdf]

Supplementary Material

1 Supplementary Tables

Table A1. Descriptive statistics of sociodemographic and COVID-19-related variables.

**Variable**	***N***	**%**	**Mean (*SD*)**
Sex			
Male	504	29.7	
Female	1,192	70.3	
Age			
18–32 years	360	21.3	
33–47 years	621	36.7	
48–62 years	675	39.9	
Older than 62 years	34	2.0	
Care responsibilities in own household			
No	1,126	66.0	
Yes	579	34.0	
Worries about lack of (child-) care (0–5)	1,684		2.15 (2.02)
Occupation type			
Nurse	613	36.7	
Doctor	239	14.3	
Medical support	267	16.0	
Administrative staff	473	28.3	
Other	80	4.8	
Completed pandemic training			
No	783	46.0	
Yes	918	54.0	
Perceived risk of infection (0–5)	1,706		2.41 (1.71)

Table A2. Determinants of transformation perceptions on the individual, team, and organizational level including control variables.

Variable	Model 1 (DV = Individual Transformation)	Model 2 (DV = Team Transformation)	Model 3 (DV = Organizational Transformation)
Individual resilience	0.18***	0.05	0.05*
Team efficacy	0.08**	0.27***	-0.01
Organizational resilience	0.21***	0.25***	0.55***
Age	0.01	-0.08***	-0.06**
Sex (0 = male, 1 = female)	0.002	-0.06**	-0.02
Perceived risk of infection	-0.09***	0.04	-0.10***
Care responsibilities in own household (0 = no, 1 = yes)	0.02	0.02	0.04
Worries about lack of (child-) care	-0.06*	0.01	-0.04
Completed pandemic training (0 = no, 1 = yes)	-0.02	0.07**	0.05**
Observations	1,617	1,616	1,617
R^2	0.17	0.23	0.37
Adjusted R^2	0.17	0.22	0.37

Note. Regression results (standardized beta coefficients) from multiple linear regression models with control variables. Individual transformation was the dependent variable in Model 1, team transformation in Model 2, and organizational transformation in Model 3. DV = dependent variable. Significant at level * $p < .05$, ** $p < .01$, *** $p < .001$.

Table A3. Results of sequential mediation analyses focusing on individual transformation.

**Outcome Model 1: Team Efficacy**					
	**Coefficient**	***SE***	***t***	***p***	***R²***
					0.15
Individual Resilience	0.47	0.03	14.59	< .001	
**Outcome Model 2: Organizational Resilience**					
	**Coefficient**	***SE***	***t***	***p***	***R²***
					0.30
Individual Resilience	0.20	0.02	8.11	< .001	
Team Efficacy	0.36	0.02	17.90	< .001	
**Outcome Model 3: Individual transformation**					
	**Coefficient**	***SE***	***t***	***p***	***R²***
					0.16
Individual Resilience (direct effect (c'))	0.14	0.02	7.01	< .001	
Team Efficacy	0.05	0.02	2.80	.005	
Organizational Resilience	0.18	0.02	7.81	< .001	
**Outcome Model 4: Individual transformation**					
	**Effect**	***SE***	***t***	***p***	***R²***
					0.09
Total effect of Individual Resilience on Individual transformation (c)	0.23	0.02	12.05	< .001	
**Indirect effect of Individual Resilience on Individual transformation**					
	**Effect**	**Boot SE**	**Boot Lower 95% CI**	**Boot Upper 95% CI**	
Individual Resilience → Team Efficacy → Organizational Resilience → Individual transformation	0.03	0.005	0.02	0.04	

Note. Analyses conducted with PROCESS Model 6 in SPSS.

Y = Individual transformation
X = Individual Resilience
M1 = Team Efficacy
M2 = Organizational Resilience
Sample Size: N = 1683

Table A4: Results of sequential mediation analyses focusing on Team transformation.

**Outcome Model 1: Team Efficacy**					
	**Coefficient**	***SE***	***t***	***p***	**R^2**
					0.15
Individual Resilience	0.47	0.03	14.59	< .001	
**Outcome Model 2: Organizational Resilience**					
	**Coefficient**	***SE***	***t***	***p***	**R^2**
					0.30
Individual Resilience	0.20	0.02	8.10	< .001	
Team Efficacy	0.36	0.02	17.90	< .001	
**Outcome Model 3: Team transformation**					
	**Coefficient**	***SE***	***t***	***p***	**R^2**
					0.21
Individual Resilience (direct effect (c'))	0.03	0.02	1.95	.051	
Team Efficacy	0.15	0.02	9.11	< .001	
Organizational Resilience	0.16	0.02	7.64	< .001	
**Outcome Model 4: Team transformation**					
	**Effect**	***SE***	***t***	***p***	**R^2**
					0.06
Total effect of Individual Resilience on Team transformation (c)	0.16	0.02	9.46	< .001	
**Indirect effect of Individual Resilience on Team transformation**					
	**Effect**	**Boot SE**	**Boot Lower 95% CI**	**Boot Upper 95% CI**	
Individual Resilience → Team Efficacy → Organizational Resilience → Team transformation	0.03	0.004	0.02	0.04	

Note. Analyses conducted with PROCESS Model 6 in SPSS.

Y = Team transformation X = Individual Resilience
M1 = Team Efficacy M2 = Organizational Resilience
Sample Size: N = 1682

Table A5: Results of sequential mediation analyses focusing on Organizational transformation.

**Outcome Model 1: Team Efficacy**					
	**Coefficient**	***SE***	***t***	***p***	R^2
					0.15
Individual Resilience	0.47	0.03	14.58	< .001	
**Outcome Model 2: Organizational Resilience**					
	**Coefficient**	***SE***	***t***	***p***	R^2
					0.30
Individual Resilience	0.20	0.02	8.10	< .001	
Team Efficacy	0.36	0.02	17.93	< .001	
**Outcome Model 3: Organizational transformation**					
	**Coefficient**	***SE***	***t***	***p***	R^2
					0.36
Individual Resilience (direct effect (c'))	0.04	0.02	2.52	.012	
Team Efficacy	0.01	0.02	0.05	.961	
Organizational Resilience	0.45	0.02	22.63	< .001	
**Outcome Model 4: Organizational transformation**					
	**Effect**	***SE***	***t***	***p***	R^2
					0.07
Total effect of Individual Resilience on Organizational transformation (c)	0.21	0.02	10.84	< .001	
**Indirect effect of Individual Resilience on Organizational transformation**					
	**Effect**	**Boot SE**	**Boot Lower 95% CI**	**Boot Upper 95% CI**	
Individual Resilience → Team Efficacy → Organizational Resilience → Organizational transformation	0.08	0.01	0.06	0.09	

Note. Analyses conducted with PROCESS Model 6 in SPSS.
Y = Organizational transformation X = Individual Resilience
M1 = Team Efficacy M2 = Organizational Resilience
Sample Size: N = 1683
